# Case report: Unilateral pulmonary artery agenesis and Kommerell's diverticulum in 1-year old girl

**DOI:** 10.3389/fped.2023.1212341

**Published:** 2023-07-31

**Authors:** Valdone Miseviciene, Gintare Liakaite, Jurgita Zaveckiene, Ausra Snipaitiene

**Affiliations:** ^1^ Pediatric Department, Lithuanian University of Health Sciences, Medical Academy, Kaunas, Lithuania; ^2^ Department of Radiology, Lithuanian University of Health Sciences, Medical Academy, Kaunas, Lithuania

**Keywords:** Kommerell’s diverticulum, rare vascular anomalies, unilateral pulmonary artery agenesis, pulmonary hypertension, pediatric vascular anomalies

## Abstract

**Background:**

Unilateral pulmonary artery agenesis (UPAA) and Kommerell's diverticulum (KD) are two rare embryologically unrelated congenital vascular malformations rarely diagnosed in children. This is the first report of our knowledge of the unique combination for a child as patients are at a high risk of pulmonary hypertension and rupture of the diverticulum. Our aim is to present the case of a pediatric patient with UPAA and KD with the short literature review and to highlight the importance of early diagnostics of rare congenital vascular malformations.

**Case report:**

A 1-year-old girl presented to the emergency department with prolonged cough and variable wheezing. A hypoplastic left lung was suspected in the radiographic image of the chest. A transthoracic echocardiogram revealed absence of the left pulmonary artery and right arch of aorta and anomaly of subclavian arteries was suspected. The diagnosis was confirmed by computed tomography scans of the chest that demonstrated elongation of the aorta and an aberrant right subclavian artery with KD, as well as absence of the left pulmonary artery. The patient is being followed up for the development of pulmonary hypertension and compression of vascular structures to the airways as well as any indications for surgical intervention because of the KD.

**Conclusions:**

UPAA and KD are two very rare congenital vascular anomalies usually diagnosed in adults. A high risk of pulmonary hypertension and rupture of diverticulum is noted for adult patients. This case provides us with an exclusive possibility to follow up a patient with an extremely rare combination of the two vascular anomalies with insufficiently known future complications and outcomes.

## 1. Introduction

A combination of the vascular malformations unilateral pulmonary artery agenesis (UPAA) and Kommerell's diverticulum (KD) is unique as patients are at a high risk of pulmonary hypertension and rupture of the diverticulum (1–3). We report a case of a pediatric patient with post-viral prolonged wheezing who was diagnosed with UPAA and KD. To the best of our knowledge, this is the first report of such a double malformation diagnosed in a pediatric patient.

## 2. Case description

**Figure 1:**
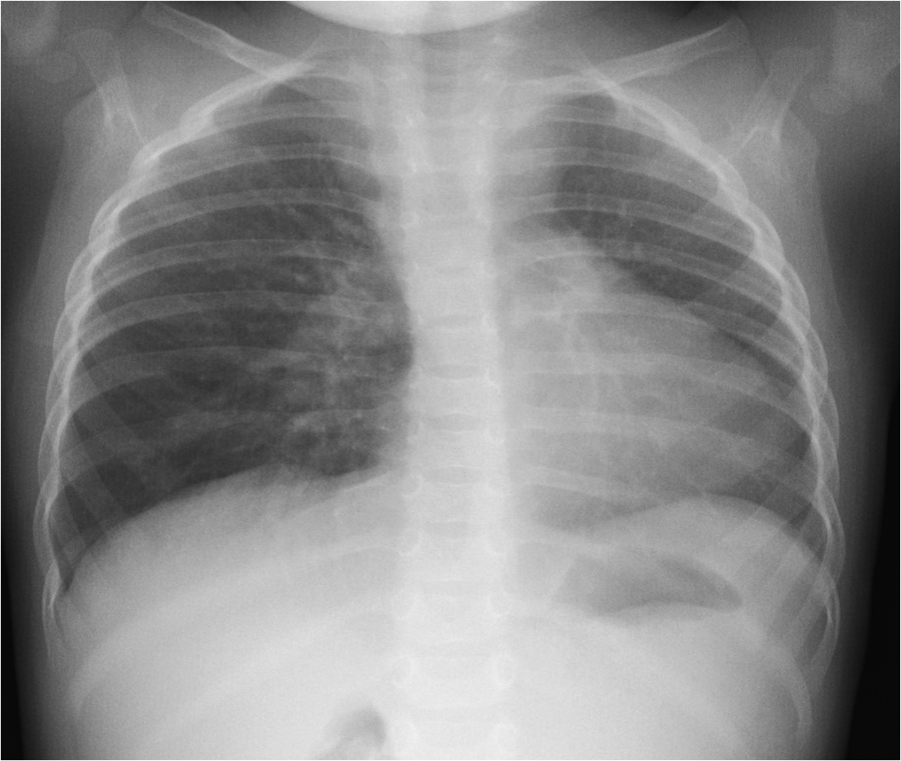
Chest radiograph image: decreased volume of the left lung, shifting of the mediastinum to the left, enlargement of the right lung hilum.

A 1-year-old girl presented to the emergency department with cough and variable wheezing, which started as an upper respiratory tract infection and persisted for two weeks after the other symptoms of the infection had resolved. She was otherwise healthy. No specific details of the personal or family history were clarified. The patient did not have tachypnea or tachycardia; pulse oximetry was normal on room air, but she had mild to moderate wheezing, expressed mainly during physical activity and not observed during sleep. Lung auscultation revealed bilateral crackles and variable, high-pitched, whistling sounds during exhalation. No other pathological findings were noted on clinical examination. Inhaled salbutamol was ineffective. Chest radiographs showed a decreased volume of the left lung, shifting of the mediastinum to the left, and enlargement of the right lung hilum (Figure 1).

**Figure 2:**
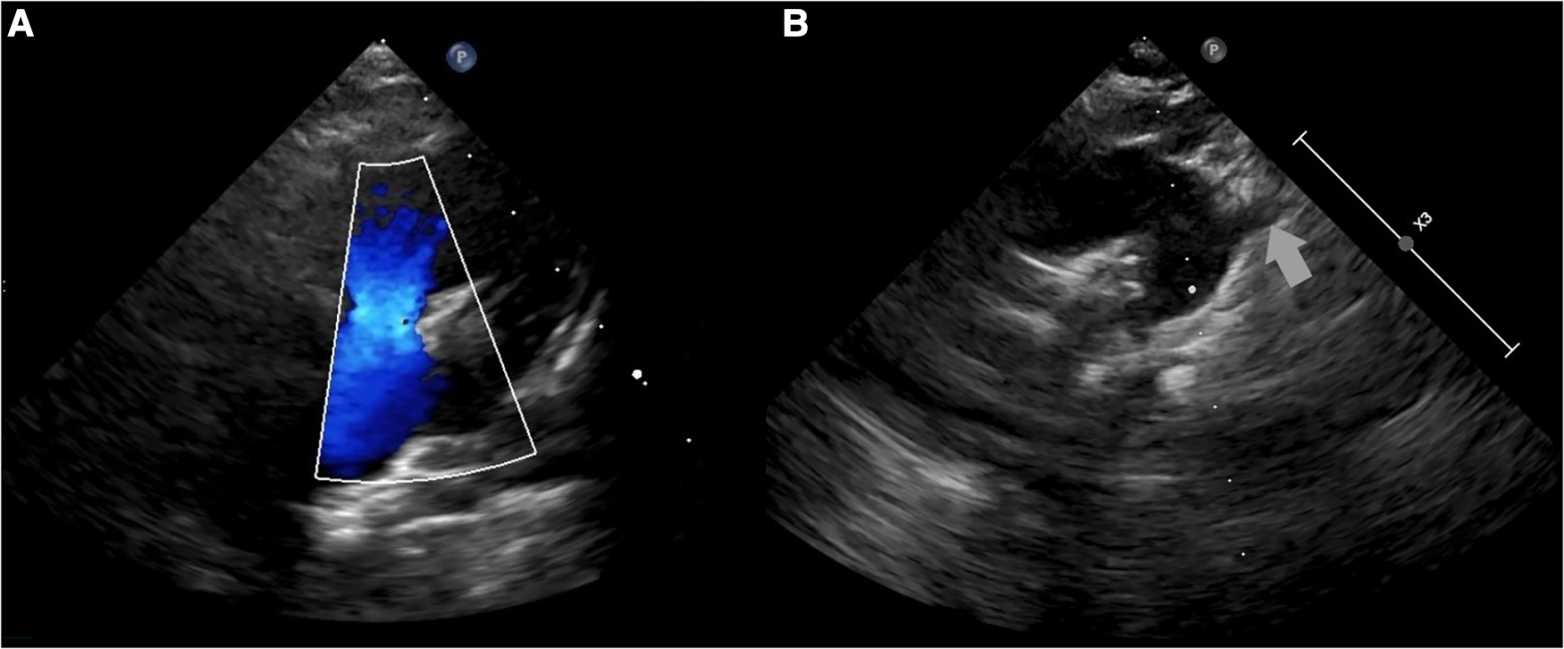
A transthoracic echocardiogram: (**A**) color Doppler flow not registered in the left pulmonary artery; (**B**) suspected right arch of aorta and anomaly of subclavian arteries.

**Figure 3:**
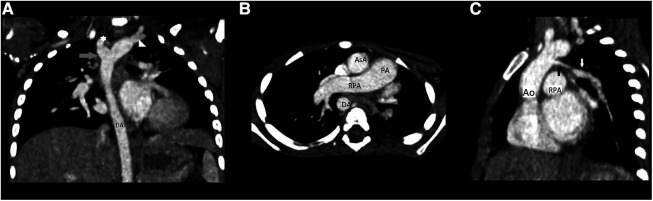
(**A**) Chest CT angiography scan, coronary reconstruction: elongation of the aorta and an aberrant right subclavian artery with KD; (**B**) chest CT angiography scan: absence of the left pulmonary artery; (**C**) chest CT angiography reconstruction: atypical vascular branches dividing from the aorta and forming the vasculature of the left lung; Abb.: Ao, aorta; AsA, ascending aorta; DA, descending aorta; PA, pulmonary artery; RPA, right pulmonary artery; star, abberant right subclavian; triangle, left subclavian artery; grey arrow, KD; black arrow, atypical branch; white arrow, anomalous left lung vascular bundle.

The patient was admitted to the pediatric department for further diagnostic approach. Wheezing persisted for two days and subsided without any specific treatment. A transthoracic echocardiogram revealed absence of the left pulmonary artery, and right arch of aorta and anomaly of subclavian arteries was suspected (Figure 2). No other congenital heart defects or signs of pulmonary hypertension were noted. The diagnosis was confirmed by computed tomography (CT) scans of the chest that demonstrated elongation of the aorta and an aberrant right subclavian artery with Kommerell's diverticulum (KD) (Figure 3A), as well as absence of the left pulmonary artery (Figure 3B), with a hypoplastic upper lobe of the left lung. Atypical vascular branches dividing from the aorta and forming the vasculature of the left lung were also visible (Figure 3C, Supplementary File S1). The mediastinum was slightly shifted to the left. We suspect that atypical vascular branches are aortopulmonary collaterals. Flow supply through ductus arteriosus to the atypical hilar segment of left pulmonary artery could be suspected as well but only angiography could specify the exact anatomy of those branches. We clarified that there is no compression to the airways of vasculature structures during bronchoscopy.

The patient is being followed up for the development of pulmonary hypertension and compression of vascular structures to the airways as well as any indications for surgical intervention because of the KD. Angiography is planned in the future to determine the exact position of the left lung arteries. During follow-up period, the patient had mild COVID-19 infection and was successfully treated at home. No specific changes were observed in the x-ray images.

## 3. Discussion

Unilateral pulmonary artery agenesis (UPAA) is a rare congenital anomaly with a prevalence of 1 in 200,000 patients (1–3). It occurs during the development of the ventral bud of the sixth aortic arch on the affected side and is usually associated with other cardiovascular anomalies (1–4). KD is the dilation of the left or right aberrant subclavian artery; its prevalence variates between 0.05% and 2% in the population (5–7). It is considered to result from incomplete regression of the fourth pharyngeal arch artery (5). Various types of KD have been reported in the literature (5, 7, 8). A vascular anomaly with an aberrant right subclavian artery has been reported as a rare type of KD, but KD in combination with left UPAA is particularly rare and there are just a few adult cases published so far (2, 8, 9). We report a case of a pediatric patient with these two rare vascular anomalies.

Left-sided UPAA is usually diagnosed during the first year of life, owing to its association with significant cardiovascular anomalies. Right-sided agenesis of the pulmonary artery is usually seen as an isolated anomaly (3). Therefore, patients without other significant anomalies can survive until adulthood without symptoms, which is the reason for the late diagnosis of UPAA. Dyspnea, limited exercise tolerance, recurrent pulmonary infections, and hemoptysis are the most common symptoms observed in adult patients with UPAA (1, 2, 4). Pulmonary hypertension develops in 19%–44% of adult patients and reduces long-term survival (1, 2, 10). There are no studies on the predicted development time of pulmonary hypertension because of late diagnosis and the inability to follow up these patients. KD is also asymptomatic in the majority of cases (2, 5, 6). Once in a while, wheezing and dysphagia could be observed because of compression of the mediastinal structures in middle-aged patients (3, 6). In our case, wheezing started together with the signs of a viral infection but because of the prolonged course of wheezing the compression of the airways was suspected. However, according to the chest CT scans, there was no compression of the airways yet. In addition, no signs of pulmonary hypertension were observed.

Heart and mediastinal displacement, ipsilateral elevation of the diaphragm, and contralateral hyperinflation are observed on chest radiographs in patients with isolated UPAA, whereas KD can only be diagnosed after chest CT or magnetic resonance imaging (MRI) (1, 3–6). Similar abnormalities of UPAA on chest radiography were observed in our patient. An abnormal x-ray scan led to a detailed examination. A transthoracic echocardiogram revealed the absence of the left pulmonary artery and was suspicious for malformations of the aortic arch vessels. Other researchers also reported cases of asymptomatic UPAA incidentally suspected after abnormal findings in the chest x-ray image (3, 4). A contrast-enhanced chest CT scan was sufficient to confirm the diagnosis of left UPAA, and it enabled us to detect an asymptomatic, extremely rare type of KD (1, 3, 4). Chest MRI or ventilation-perfusion scintigraphy can also be used to diagnose UPAA (1).

There is no specific treatment for asymptomatic UPAA, whereas KD can be treated surgically (1, 5–7, 11). Some authors suggest surgical treatment for asymptomatic KD larger than 5 cm (or even 3 cm), while others advocate that all KD should be surgically treated, regardless of size (3, 5–7, 11). It is noted, that there is no data about surgical options in asymptomatic infants and children and all reported surgical treatment cases are about adult patients. Undoubtedly all patients with symptomatic KD should undergo surgery. Sildenafil is recommended for the treatment of pulmonary hypertension (10). Also, patients with UPAA and pulmonary hypertension should undergo surgical reconstruction and pulmonary artery rehabilitation (12). Pulmonary angiography and embolization can be performed in patients with hemoptysis because of the chronically hyperperfused vessels on the contralateral side of the UPAA (1, 4, 13). The lung without a pulmonary artery usually plays a minor role in gas exchange, and pneumonectomy could be suggested in the future (1–4). Our patient had not yet undergone surgical treatment. She is currently on follow-up for possible dilation of the KD and signs of pulmonary hypertension.

## 4. Conclusions

UPAA and KD are two rare, embryologically unrelated congenital vascular anomalies usually diagnosed in adults. Careful consideration of abnormal findings in chest x-ray lead us to detailed investigation and early diagnosis of two rare vascular malformations. Both anomalies can be assessed with chest CT scans, while chest MRI can also be used for diagnostic purposes without radiation. There is no consensus on the treatment or follow-up recommendations, especially for pediatric patients. Each patient has to be managed individually. This case provides us with an exclusive possibility to follow up a patient with an especially rare combination of the two vascular anomalies with insufficiently known future complications and outcomes.

## Data Availability

The raw data supporting the conclusions of this article will be made available by the authors, without undue reservation.

## Supplementary material

Supplementary File 1 Three-dimensional computed tomography (3D CT) reconstruction images of the heart and great vessels.

## References

[1] Steiropoulos P Archontogeorgis K Tzouvelekis A Ntolios P Chatzistefanou A Bouros D. Unilateral pulmonary artery agenesis: a case series. Hippokratia. (2013) 17(1):73–6. https://www.ncbi.nlm.nih.gov/pmc/articles/PMC3738284/23935349 PMC3738284

[2] Rousou AJ Tetenta S Boffa DJ. Pulmonary artery agenesis and Kommerell's Diverticulum presenting with hemoptysis. Eur J Cardiothorac Surg. (2009) 35:370–2. 10.1016/j.ejcts.2008.10.053 19109028

[3] Aypak C Yıkılkan H Uysal Z Görpelioğlu S. Unilateral absence of the pulmonary artery incidentally found in adulthood. Case Rep Med. (2012) 2012:942074. 10.1155/2012/942074 22685472 PMC3368213

[4] Weldetsadik AY Asfaw YM Tekleab AM. Isolated absence of right pulmonary artery in a 4-year old child: a case report. Int Med Case Rep J. (2018) 11:297–301. 10.2147/IMCRJ.S181571. 30464650 PMC6214340

[5] Rosendael PJ Stöger JL Kiès P Vliegen HW Hazekamp MG Koolbergen DR The clinical Spectrum of Kommerell's Diverticulum in adults with a right-sided aortic arch: a case series and literature overview. J Cardiovasc Dev Dis. (2021) 8:25. 10.3390/jcdd8030025 33652796 PMC7996811

[6] Robles TA Srinivasan A Mazur L Gourishankar A. Kommerell's diverticulum with a twist: a case of recurrent wheeze in an 8-year-old boy. Global Pediatric Health. (2019) 6:1–4. 10.1177/2333794X19897506 PMC6926987 31903415

[7] Bhatt TC Muralidharan CG Singh G Jain NK. Kommerell's diverticulum: a rare aortic arch anomaly. Med J Armed Forces India. (2016) 72(1):S80–3. 10.1016/j.mjafi.2016.09.003 28050078 PMC5192231

[8] Sahin H Sarioglu FC Pekcevik Y Akay E Çapar AE Oztekin O The kommerell diverticulum revisited: embryogenesis, imaging findings of various types and clinical implications. ECR Congress. (2016). Poster no.: C-1401. 10.1594/ecr2016/C-1401. https://epos.myesr.org/poster/esr/ecr2016/C-1401

[9] Hassan W Omrani AA Neimatallah M Fadley FA Halees ZA. Dysphagia lusoria caused by aberrant right subclavian artery, Kommerell's Diverticulum, legamentum ring, right descending aorta, and absent left pulmonary artery: a report of a unique vascular congenital disease undetected until adulthood and a review of the literature. Pediatr Cardiol. (2005) 26(6):851–5l. 10.1007/s00246-005-0936-1 16088417

[10] Rodríguez-Gómez F Martin I Sánchez A Pujol E. Sildenafil treatment of unilateral pulmonary edema and pulmonary hypertension in pulmonary artery agenesis. Rev Esp Cardiol. (2006) 59(12):1345–5. 10.1157/13096595 17194435

[11] Erben Y Brownstein AJ Velasquez CA Li Y Rizzo JA Mojibian H Natural history and management of Kommerell's Diverticulum in a single tertiary referral center. J Vasc Surg. (2020) 71(6):2004–11. 10.1016/j.jvs.2019.08.260 31708305

[12] Yan X Cen J Luo X Chen J Wen S Wu J Surgical repair of unilateral absence of pulmonary artery in children with pulmonary hypertension: a single-center retrospective study. Transl Pediatr. (2022) 11(11):1813–22. 10.21037/tp-22-491 36506767 PMC9732598

[13] Johnson TRC Thieme SF Deutsch MA Hinterseer M Reiser MF Becker CR Unilateral pulmonary artery agenesis. Noninvasive diagnosis with dual-source computed tomography. Circulation. (2009) 119(8):1158–60. 10.1161/CIRCULATIONAHA.108.777698 19255355

